# Supplementary material for: Incrimination of shrews as a reservoir for Powassan virus
Source: Commun Biol. 2021 Nov 22;4:1319. doi: 10.1038/s42003-021-02828-1 (PMC8608897; doi:10.1038/s42003-021-02828-1)
Supplement: Supplementary file 2 — Reporting Summary [file 42003_2021_2828_MOESM2_ESM.pdf]

nature portfolio

Corresponding author(s): Heidi Goetehrt

Last updated by author(s): Oct 25, 2021

Reporting Summary

Nature Portfolio wishes to improve the reproducibility of the work that we publish. This form provides structure for consistency and transparency in reporting. For further information on Nature Portfolio policies, see our Editorial Policies and the Editorial Policy Checklist.

Statistics

For all statistical analyses, confirm that the following items are present in the figure legend, table legend, main text, or Methods section.

n/a	Confirmed	
☐	☒	The exact sample size (n) for each experimental group/condition, given as a discrete number and unit of measurement
☐	☒	A statement on whether measurements were taken from distinct samples or whether the same sample was measured repeatedly
☐	☒	The statistical test(s) used AND whether they are one- or two-sided<br>*Only common tests should be described solely by name; describe more complex techniques in the Methods section.*
☐	☒	A description of all covariates tested
☒	☐	A description of any assumptions or corrections, such as tests of normality and adjustment for multiple comparisons
☐	☒	A full description of the statistical parameters including central tendency (e.g. means) or other basic estimates (e.g. regression coefficient) AND variation (e.g. standard deviation) or associated estimates of uncertainty (e.g. confidence intervals)
☐	☒	For null hypothesis testing, the test statistic (e.g. F, t, r) with confidence intervals, effect sizes, degrees of freedom and P value noted<br>*Give P values as exact values whenever suitable.*
☒	☐	For Bayesian analysis, information on the choice of priors and Markov chain Monte Carlo settings
☒	☐	For hierarchical and complex designs, identification of the appropriate level for tests and full reporting of outcomes
☐	☒	Estimates of effect sizes (e.g. Cohen's d, Pearson's r), indicating how they were calculated

Our web collection on statistics for biologists contains articles on many of the points above.

Software and code

Policy information about availability of computer code

Data collection	*Provide a description of all commercial, open source and custom code used to collect the data in this study, specifying the version used OR state that no software was used.*
Data analysis	*Provide a description of all commercial, open source and custom code used to analyse the data in this study, specifying the version used OR state that no software was used.*

For manuscripts utilizing custom algorithms or software that are central to the research but not yet described in published literature, software must be made available to editors and reviewers. We strongly encourage code deposition in a community repository (e.g. GitHub). See the Nature Portfolio guidelines for submitting code & software for further information.

Data

Policy information about availability of data

All manuscripts must include a data availability statement. This statement should provide the following information, where applicable:

- Accession codes, unique identifiers, or web links for publicly available datasets
- A description of any restrictions on data availability
- For clinical datasets or third party data, please ensure that the statement adheres to our policy

Sequences from this study have been deposited into GenBank (Accession # MZ148230-MZ148271). All other data has been deposited in OSF and can be accessed at https://osf.io/5dzyp/?view_only=7b9dfd42baf2472aa802cb88efbc94d1. The map was adapted from an open source website (http://www.amaps.com/mapstoprint/LIST%20OF%20states.htm).

Field-specific reporting

Please select the one below that is the best fit for your research. If you are not sure, read the appropriate sections before making your selection.

☐ Life sciences ☐ Behavioural & social sciences ☒ Ecological, evolutionary & environmental sciences

For a reference copy of the document with all sections, see nature.com/documents/nr-reporting-summary-flat.pdf

Ecological, evolutionary & environmental sciences study design

All studies must disclose on these points even when the disclosure is negative.

Study description	Collected ticks from field sites in Massachusetts and Rhode Island examined for infection with deer tick virus and B. burgdorferi and then determined what the ticks had fed on using bloodmeal analysis.
Research sample	Ticks were collected from 12 different field sites. Trapping for shrews took place a 2 sites.
Sampling strategy	These were convenience samples.
Data collection	Ticks were counted and extracted for DNA back in the lab.
Timing and spatial scale	Collections occurred during the summers of 2018-2020.
Data exclusions	none
Reproducibility	N/A
Randomization	N/A
Blinding	N/A

Did the study involve field work? ☐ Yes ☐ No

Field work, collection and transport

Field conditions	Ticks were collected on dry days during the months of June and July.
Location	Collections occurred on Naushon Island, Martha's Vineyard and Nantucket, as well as 2 sites in Washington County in Rhode Island.
Access & import/export	N/A
Disturbance	N/A

Reporting for specific materials, systems and methods

We require information from authors about some types of materials, experimental systems and methods used in many studies. Here, indicate whether each material, system or method listed is relevant to your study. If you are not sure if a list item applies to your research, read the appropriate section before selecting a response.

Materials & experimental systems

n/a	Involved in the study	
☒	☐	Antibodies
☒	☐	Eukaryotic cell lines
☒	☐	Palaeontology and archaeology
☐	☒	Animals and other organisms
☒	☐	Human research participants
☒	☐	Clinical data
☒	☐	Dual use research of concern

Methods

n/a	Involved in the study	
☒	☐	ChIP-seq
☒	☐	Flow cytometry
☒	☐	MRI-based neuroimaging

Animals and other organisms

Policy information about studies involving animals; ARRIVE guidelines recommended for reporting animal research

Laboratory animals	CD-1 mice

Wild animals	shrews
Field-collected samples	Ticks were stored at -20C, shrews were killed in the field and tissues were stored at -20C.
Ethics oversight	Our use of animals has been covered under existing IACUC approvals (Tufts University, G2020-101, expiration 2/2023).

Note that full information on the approval of the study protocol must also be provided in the manuscript.
